# A Great City's Hospital Work

**Published:** 1914-07-11

**Authors:** 


### The Hospital

The Workers' Newspaper of Administrative Medicine and Institutional Life, Administration, National Insurance and Health.

No. 1464, Vol. LVI. SATURDAY, JULY 11, 1914. PRICE ONE PENNY.

### A GREAT CITY'S HOSPITAL WORK.

Their Majesties the King and Queen have had many interesting experiences since King George V. ascended the throne. The King especially is genuinely and deeply interested in the maintenance, prosperity, and development of the voluntary hospitals of this country. Tuesday last must have proved to their Majesties a day to remember, for they had the privilege of opening two splendid hospitals of the first importance to the city of Glasgow. To their Majesties the opening of a new hospital is no mere formal ceremony. They both take the deepest interest in the character and quality of the institutions which they honour with their presence. The King has by hard work and constant visitation acquired an exceptional knowledge of hospital construction and administration. We can well understand how gratified and surprised many of the Glasgow managers were at the proofs their Majesties gave during their visit to the two great hospitals referred to of their Majesties' appreciation and knowledge. The opening of the Glasgow Royal Infirmary had a peculiar interest for His Majesty seeing that when Prince of Wales he went specially to Glasgow in order to lay the foundation-stone of this institution seven years ago. At that time, April 1907, King George did not inspect the old hospital buildings, for in truth they contained little that was desirable, and practically the whole of those which were standing at that date have been scrapped.

It is a remarkable fact that the managers of the Glasgow Royal Infirmary should have boldly faced the rebuilding of the whole of this hospital, and so arranged matters that the work of the institution has been carried on with efficiency during the seven years it has taken to complete the work. Owing to the special care and business aptitude displayed by the managers, under the chairmanship of Mr. J. D. Hedderwick, LL.D., especial pains were taken, as we explained last week (page 367), to secure that not only the plans, but the whole of the internal arrangements should be entrusted to three experts, so that the new Royal Infirmary, opened on Tuesday last, has necessarily a peculiar interest for all hospital workers, architects, and hospital managers all the world over. We have no doubt that His Majesty King George was fully acquainted with these facts, and that this knowledge made the opening ceremony and inspection of the new hospital a source of real interest to him. This is a fine institution, and is one of the best and largest of the voluntary hospitals. Queen Mary, whose devotion to the interests of children has made her beloved by every mother throughout the British Empire, naturally took a supreme interest in the new Royal Hospital for Sick Children. We regret there was no time for their Majesties to inspect the Western Infirmary, but it was a signal mark of Royal favour that they should have found time to visit it on so busy a day. It is so extraordinarily well-managed as to make it just the sort of hospital King George would find pleasure in inspecting. It was a memorable event for the King and Queen to pay a visit to Glasgow for the purposes we have just referred to in the busiest month of the season. We hope that the enthusiastic loyalty with which they were received will fill the Glasgow visit with pleasant memories for their Majesties.

Glasgow has long aspired to be regarded as the second city in the United Kingdom, and the mere recital of the enormous sums spent upon the two hospitals, which were opened by their Majesties on this day, proves eloquently the deep hold the voluntary hospital system has upon Scotsmen as well as Englishmen. Scotsmen do not part with their money for nought, and it is indeed splendid evidence of a deep conviction that practically the whole of the money required to build and equip the Royal Infirmary and the Royal Hospital for Sick Children—approximately £700,000—had been provided prior to the date of the opening ceremony. Glasgow already has in the Western Infirmary a hospital of the first rank, full of up-to-date structural units, whose out-patient department has attracted visitors from all parts of the world. Dr. Mackintosh has proved himself to be a medical superintendent whose work constitutes a model of what a medical superintendent's should be. To Dr. Thom, the superintendent of the Glasgow Royal Infirmary, great credit is due for the fact that, during the nine years it has taken to rebuild this large hospital, containing 665 beds, the ordinary work of this great institution has been carried on with marked efficiency, without any complaint from the patients or their friends. The managers of the Royal Infirmary are entitled to feel proud that their long and arduous labours have culminated in the provision of so fine a hospital, and Mr. Hedderwick, their chairman, whose services were detailed in The Hospital of June 27, 1914, page 343, will, we hope, receive some adequate recognition of his public services, for he has been throughout the life and soul of one of the greatest hospital enterprises which have been undertaken and completed in recent years. Glasgow's citizens have always been intensely proud of their city, and the occasion which brought their Majesties the King and Queen to Glasgow on the 7th instant should make them, if possible, prouder than ever.

